# Effects of cadmium contamination on bacterial and fungal communities in *Panax ginseng*-growing soil

**DOI:** 10.1186/s12866-022-02488-z

**Published:** 2022-03-19

**Authors:** Hai Sun, Cai Shao, Qiao Jin, Meijia Li, Zhenghai Zhang, Hao Liang, Huixia Lei, Jiaqi Qian, Yayu Zhang

**Affiliations:** 1 Institute of Special Animal and Plant Science of Chinese Academy of Agricultural Science, Changchun, 130112 China; 2 Jilin Provincial Key Laboratory of Traditional Chinese Medicinal Materials Cultivation and Propagation, Changchun, 130062 People’s Republic of China; 3 College of Pharmacy and biological Engineering, Chengdu University, Chengdu, 610106 China

**Keywords:** *Panax ginseng*, Cadmium contamination, Microbial community, HiSeq sequencing

## Abstract

**Background:**

Cadmium (Cd) contamination in soil poses a serious safety risk for the development of medicine and food with ginseng as the raw material. Microorganisms are key players in the functioning and service of soil ecosystems, but the effects of Cd-contaminated ginseng growth on these microorganisms is still poorly understood. To study this hypothesis, we evaluated the effects of microorganisms and Cd (0, 0.25, 0.5, 1.0, 2.0, 5.0, and 10.0 mg kg^-1^ of Cd) exposure on the soil microbial community using Illumina HiSeq high-throughput sequencing.

**Results:**

Our results indicated that Cd-contaminated soil affected the soil microbial diversity and composition, and bacterial diversity was affected more than fungal diversity in Cd-contaminated soil, especially according to Shannon indices. The abundance of the soil microbial community decreased and the composition changed according to the relative abundances at the phylum level, including those of *Saccharibacteria* and *Gemmatimonadetes* in bacteria and *Mortierellomycota* in fungi. The LEfSe algorithm was used to identify active biomarkers, and 45 differentially abundant bacterial taxonomic clades and 16 differentially abundant fungal taxonomic clades were identified with LDA scores higher than 4.0. Finally, a heatmap of Spearman's rank correlation coefficients and canonical discriminant analysis (CDA) indicated that some key biomarkers, *Arenimonas*, *Xanthomonadales*, *Nitrosomonadaceae*, *Methylophilales*, *Caulobacterales*, *Aeromicrobium*, *Chitinophagaceae*, *Acidimicrobiales*, *Nocardioidaceae*, *Propionibacteriales*, *Frankiales*, and *Gemmatimonadaceae,* were positively correlated with the total and available Cd (*p*<0.05) but negatively correlated with AK, AP, and pH (*p*<0.05) in the bacterial community. Similarly, in the fungal community, *Tubaria*, *Mortierellaceae*, and *Rhizophagus* were positively correlated with the total and available Cd but negatively correlated with AK, AP, TK, and pH.

**Conclusion:**

Cd contamination significantly affected microbial diversity and composition in ginseng-growing soil. Our findings provide new insight into the effects of Cd contamination on the microbial communities in ginseng-growing soil.

**Supplementary Information:**

The online version contains supplementary material available at 10.1186/s12866-022-02488-z.

## Background

Ginseng, the root and rhizome of *Panax ginseng* CA Meyer, is mainly cultivated in the northeastern China [1]. Due to its extremely high medicinal value, ginseng has been widely used to treat diseases, such as neurodegenerative diseases, cardiovascular disease, and oxidative damage diseases, for thousands of years [2–4]. Previous studies have indicated that ginseng cultivation is often threatened by the addition of fertilizer and organic fertilizer, which causes an increase in heavy metals in soils [5]. Heavy metals may cause functional disorders of soil [6], inhibit plant growth, and even endanger human health through a contaminated food chain [7, 8]. Nevertheless, understanding the mechanisms of these interactions between soil function and heavy metals remains a major challenge in soil ecology.

Soil pollution has become a prominent environmental issue in China due to the excessive use of chemical fertilizers in agricultural activities [9, 10] and air pollution from industrial emissions and vehicle exhaust [11]. Heavy metals in surface soil, such as cadmium (Cd), arsenic (As), lead (Pb), iron (Fe), copper (Cu), and zinc (Zn), can cause soil pollution [12]. Heavy metal pollution in soil is divided into two categories based on the toxicity and necessity for plant growth and development. Some heavy metals with stronger toxicity such as Cd, As and Pb, are well studied because of their persistence, toxicity and nonactive degradation. Others heavy metals are essential elements for plant growth [13], such as Fe, Cu, Zn, but too much of these will cause heavy metal pollution. Cd is one of the most hazardous elements and has been a public concern with increasing risk in soil remediation. Cd pollution poses a major threat to human and animal health due to its continuous release into the environment, especially soil, even at a low concentration.

Most of the large number of studies on heavy metal passivators in croplands focused on the effects of passivators on the proportions of heavy metals in soils and the translocation of heavy metals from soil to crops. However, few have focused on Cd contamination on the soil microbial community in *Panax ginseng*-growing soil [14]. Soil microorganisms are the most abundant and diverse life forms on earth, and they have important effects on biogeochemical processes and nutrient transformation [15, 16]. Soil microorganisms are an important index for measuring soil quality and are involved in soil organic matter decomposition, mineralization and nutrient utilization. However, they are affected by biotic and abiotic factors, such as pH, soil organic matter, and other toxic metals [17, 18]. When the accumulation of heavy metals in soil exceeds a certain range, they will cause certain toxic effects on microorganisms, and further affect the balance of the soil ecosystem or even destroy the function of the soil environmental system. As an important index representing soil functional stability and ecosystem structure, soil microbial communities can effectively monitor the soil environmental pollution status [19, 20]. The toxic mechanism may be caused by the destruction of microbial cells, changes in the enzyme system, and abruption of the normal metabolic response of cells [21, 22]. Soil microorganisms are used as an index to evaluate the soil pollution level in recently contaminated soil [23].

Cd is a nonessential element for plant growth. Its characteristics include high toxicity, nonbiodegradability and long-term accumulative behaviour, which can cause serious illnesses in humans though the food chain [24]. Cd contamination not only affects soil fertility but also disturbs the microbial community structure and reduces biodiversity [25]. Soil microbial communities can quickly respond to environmental stress in the rhizosphere and further adjust the direction of the dominant microorganism [26]. For example, Deng et al. [27] found that Cd plays a vital role in decreasing alpha diversity in a typical Cd-contaminated farmland ecosystem and that some Cd-tolerant microbes become the dominant microbes. However, other studies showed that AM fungi can adapt to Cd-contaminated soil and help plants adapt to adverse growing conditions by reshaping the microbial community structure [28]. Therefore, some microbes are regarded as environmental monitoring factors.

We investigated that the effects of different Cd contaminated levels on the microbial community structure in ginseng-growing soil, and 16S and ITS1 were analysed using Illumina HiSeq high-throughput sequencing technology. We hypothesized that: (1) the microbial abundance, diversity, and community composition might be affected by the different Cd-contaminated treatments, especially for the bacterial community, We also hypothesized that due to the directed regulation of Cd on microorganisms, biodiversity and some key biomarkers would be the dominant microbes in response to the degree of Cd contamination. Finally, we aimed to identify microbes and fungi related toCd contamination as monitoring factors and apply them in planting management.

## Results

### Total and available cadmium in soil

**Fig. 1:**
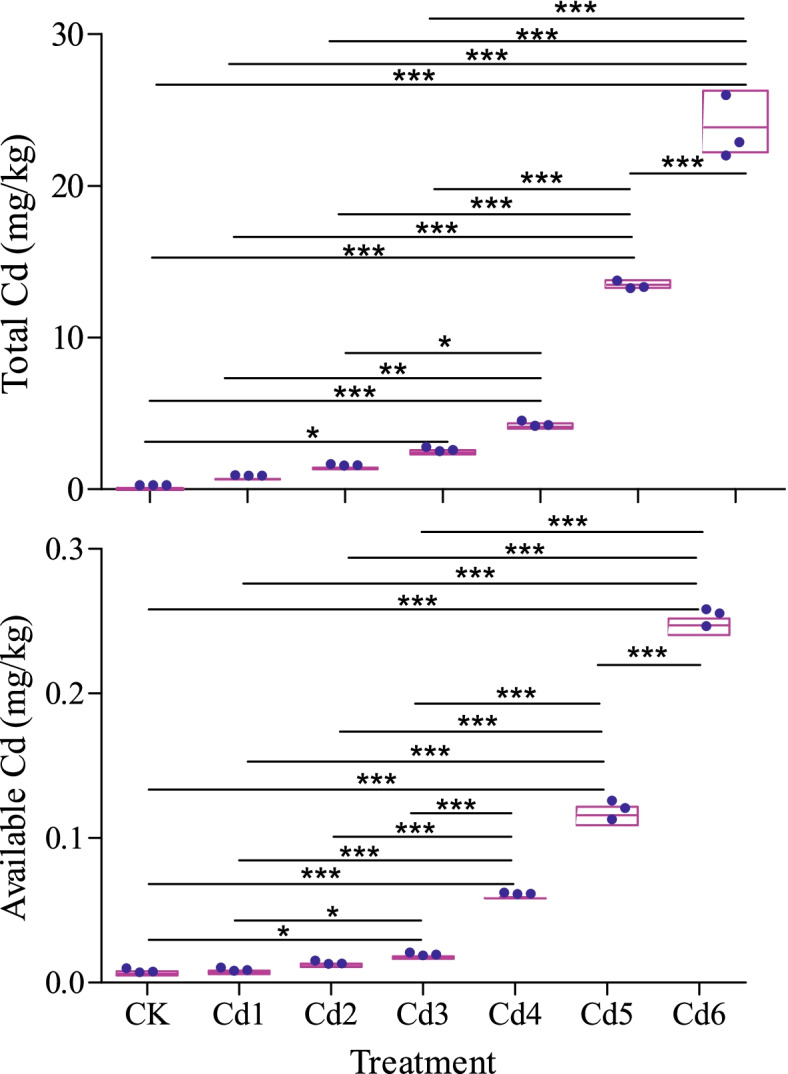
The concentration of total and available Cd in different treatment, the symbols *, ** and *** were used only to indicated significantly with *p*<0.5, *p*<0.01 and *p*<0.001, respectively

After a complete ginseng growth period, the concentration of total and available Cd was higly variable among the different treatments (Fig. 1). The addition of Cd significantly increased the total and available Cd concentrations in the soil among the seven treatment soils based on Duncan's multiple comparison (*p*<0.05 or *p*<0.001). Although the total and available Cd concentrations were not significantly increased in the Cd1 and Cd2 treatments compared with the CK group, but the total and available Cd concentrations in the Cd3, Cd4, Cd5, and Cd6 treatments groups were significantly higher than those in the CK group at the *p*<0.01 or *p*<0.001 level.

### Sequencing depth and soil microbial community diversity analysis

A total of 1400903 sequence reads in 16S were generated from 21 samples, with an average of 66710 sequences per sample. However, a total of 1678800 sequence reads in ITS1 were generated from 21 samples, with an average of 79943 sequences per sample. The average sequence lengths of these sequences are approximately 418 bps and 319 bps in 16S and ITS1, respectively. The reads were clustered into 1007 OTUs and 714 OTUs in 16S and ITS1, respectively, based on 97% sequence similarity (Fig. S1 and S2).

**Fig. 2:**
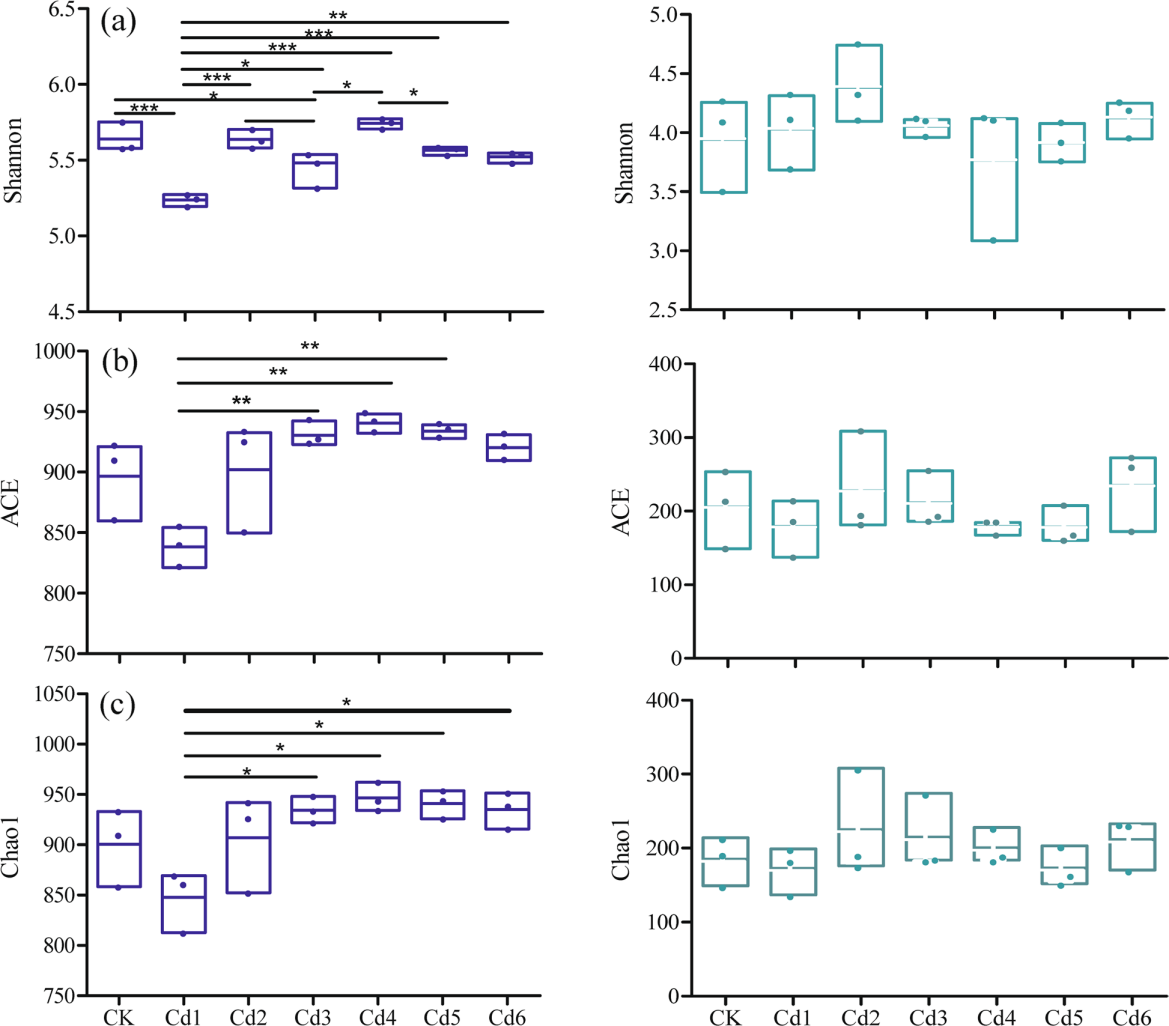
The index of Shannon, ACE and Chao1 of the microbial(a) and fungal(b) of ginseng roots in the different Cd addition treatment. The horizontal bars within boxes represent medians. The tops and bottoms of boxes represent the 75th and 25th percentiles, respectively. the symbols *, ** and *** were used only to indicated significantly with *p*<0.5, *p*<0.01 and *p*<0.001, respectively

Three α-diversity indices (Shannon, ACE and Chao1) were used to evaluate the soil microbial community richness and diversity (Fig. 2). Although cadmium addition did not significantly affect fungal taxonomic α-diversity (*p*>0.05, Fig. 2b), it had strong effects on the bacterial taxonomic α-diversity (*p*<0.05, *p*<0.01 or *p*<0.001, Fig. 2a). The Shannon index was higher in the CK group than in the Cd1 and Cd3 groups (*p*<0.001, *p*<0.05), and the index was lowest in the Cd1 group among all the treatment groups. Similarly, the ACE and Chao1 indices were lower in the Cd1 group than in the other treatment groups, especially in the Cd2, Cd3 and Cd4 treatment groups (*p*<0.05).

**Fig. 3:**
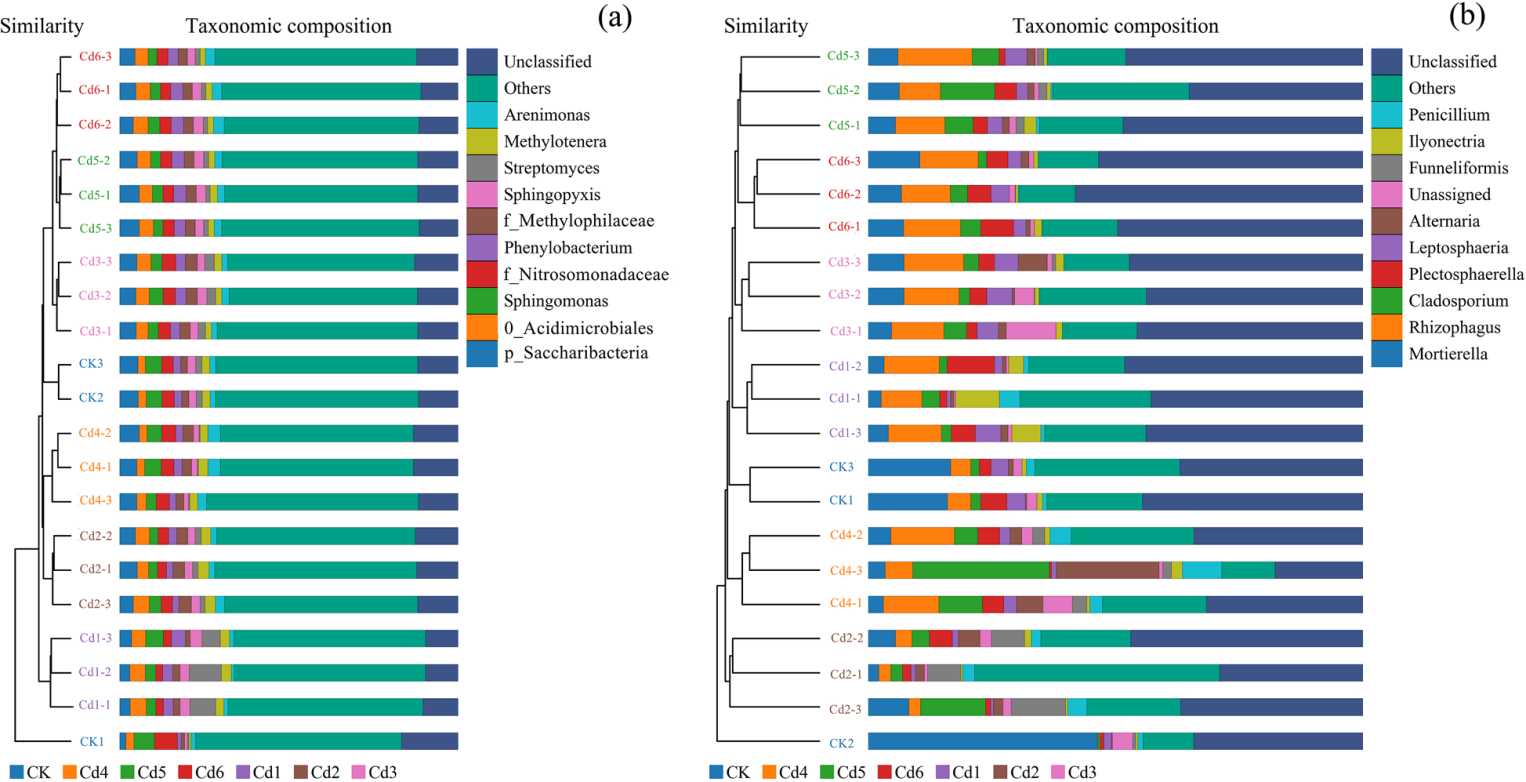
Unweighted Pair Group Method with Arithmetic Mean (UPGMA) clustering of bacterial (**a**) and fungal (**b**) communities associated with all soil samples

**Fig. 4:**
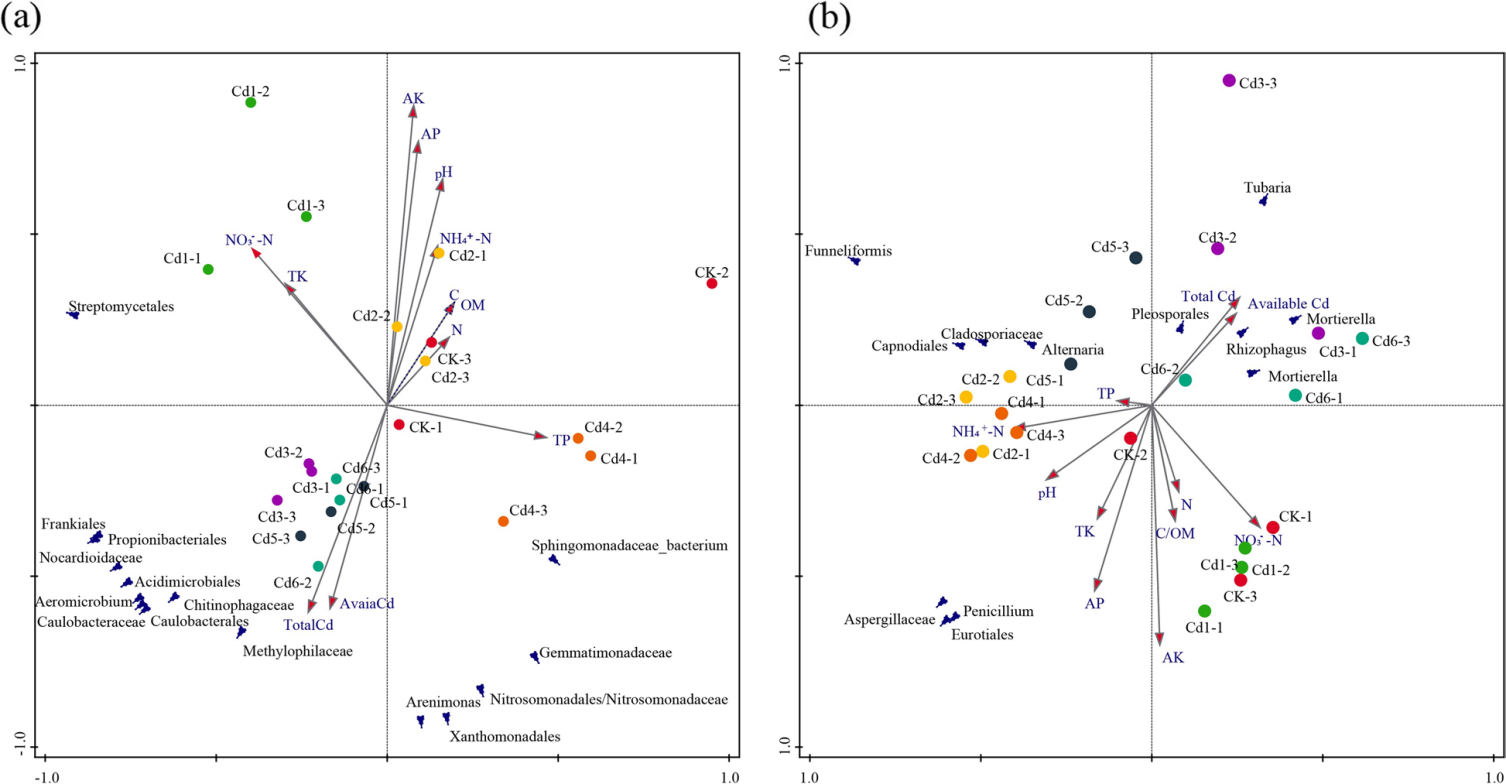
Canonical discriminant analysis biplots to investigate the ecological correlation between abundance of microorganisms and soil characteristics. Sequences of 16S(**a**) and ITS1(**b**) rDNA in the phylum level and environmental parameters

The β-diversity of the soil microbial communities in the Cd-added treatments was evaluated by UPGMA clustering analysis. The microbial communities in the seven treatments differed from each other (Fig. 3a and b), and the soil bacterial and fungal communities clustered into seven groups depending on the Cd concentration. Similarly, this grouping between bacterial and fungal communities was observed in canonical discriminant analysis (CDA) (Fig. 4). Thus, the UPGMA clustering analysis and CDA indicated that the Cd concentration might be directly correlated with the bacterial and fungal community structure.

### Correlations among microbial community, environmental parameters and phyla

The microbial community structure might be correlated with environmental parameters in the Cd-supplemented soil. Canonical discriminant analysis (CDA) was carried out on the microbes (bacteria and fungi) to link the abundance of key biomarkers with the main soil physicochemical properties (Fig. 4). The length of an environmental factor arrow indicates the strength of the environmental factors to the overall bacterial communities. Thus, AK, AP, total Cd, available Cd and NO_3_^-^-N concentrations and pH appear to be the most important environmental factors. The CDA of the relative abundance of the key biomarkers at the different classification levels and environmental factors was performed, *Arenimonas*, *Xanthomonadales*, *Nitrosomonadaceae*, *Methylophilales*, *Caulobacterales*, *Aeromicrobium*, *Chitinophagaceae*, *Acidimicrobiales*, *Nocardioidaceae*, *Propionibacteriales*, *Frankiales*, and *Gemmatimonadaceae* were positively correlated with the total and available Cd, but negatively correlated with AK, AP, and pH. Similarly, a correlation was found between fungal community structure and environmental factors, such as the abundance of *Tubaria*, *Mortierellaceae*, and *Rhizophagus*. These genera were positively correlated with the total and available Cd, but negatively correlated with AK, AP, TK and pH. The results indicated that the microbial community may be mainly regulated by the Cd concentration.

### Microbial composition in response to different treatments of cadmium addition

**Fig. 5:**
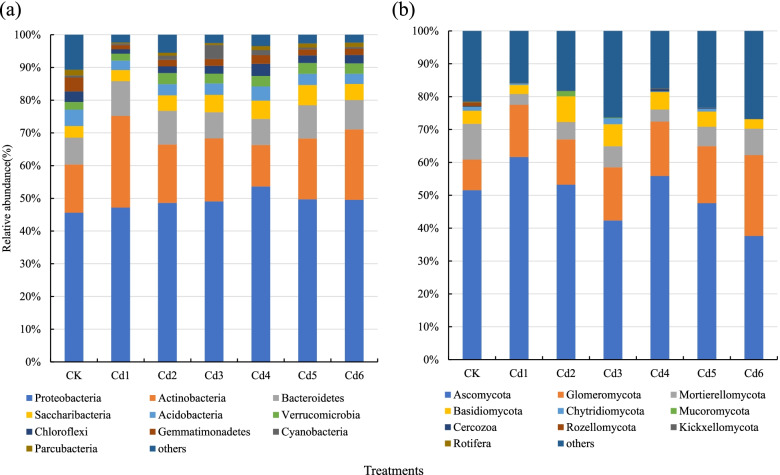
Relative abundance of bacterial (**a**) and fungal (**b**) phyla at the phylum. Only the top 10 abundance phyla are shown in this figure

The taxonomic distribution of bacteria and fungi at the phylum level is summarized in Fig. 5. Bacterial sequences were assigned to 24 phyla as shown, and *Proteobacteria* was the most abundant phylum across all samples, accounting for 45.62–53.63% of the total valid reads in all samples, with an average relative abundance of 49.05%. *Actinobacteria* was the second most abundant phylum across all samples, accounting for 12.67–28.03% of the total valid reads in all samples, with an average relative abundance of 18.94%. The other dominant phyla were *Bacteroidetes* (7.96–10.59%, averaging 9.17%), *Saccharibacteria* (3.41–6.17%, averaging 4.81%), *Acidobacteria* (2.88–5.00%, averaging 3.67%), *Verrucomicrobia* (2.10–3.38%, averaging 2.93%), *Chloroflexi* (1.36–3.72%, averaging 2.51%), *Gemmatimonadetes* (1.28–4.23%, averaging 2.28%), *Cyanobacteria* (0.50–4.29%, averaging 1.35%), and *Parcubacteria* (0.29–1.76%, averaging 1.04%). At the class level, a wide range of classes dominated. Based on the average relative abundance, the most abundant classes were *Alphaproteobacteria*, *Betaproteobacteria*, *Actinobacteria*, *Gammaproteobacteria*, and *Sphingobacteriia*.

Similarly, fungal sequences were assigned to 13 phyla as shown. *Ascomycota* was the most abundant phylum across all samples, accounting for 37.63–61.63% of the total valid reads in all samples, with an average relative abundance of 49.97%. *Glomeromycota* was the second most abundant phylum across all samples, accounting for 13.80–24.59% of the total valid reads in all samples, with an average relative abundance of 16.24%. The other dominant phyla were *Mortierellomycota* (3.30–10.89%, averaging 6.21%) and *Basidiomycota* (2.76–7.80%, averaging 4.88%). At the class level, a wide range of classes dominated. Based on the average relative abundance, the most abundant classes were *Sordariomycetes*, *Dothideomycetes*, *Glomeromycetes*, *Mortierellomycetes*, and *Leotiomycetes*.

**Fig. 6:**
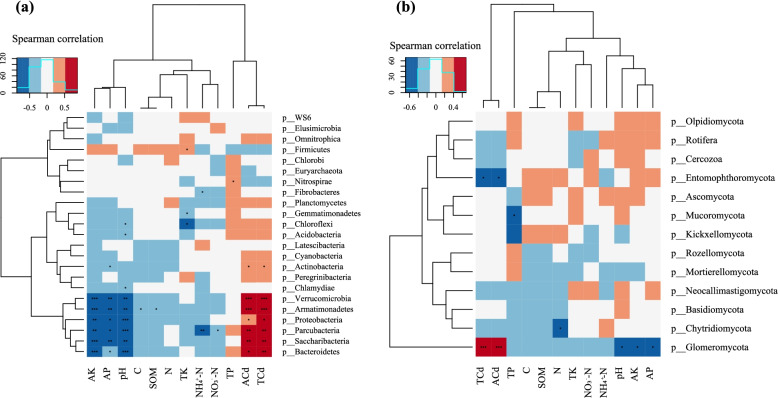
Heatmap of Spearman's rank correlation coefficients between the soil environmental variables and the relative abundance of bacterial (**a**) and fungal (**b**) communities at the phylum level, respectively. Note: Horizontal row represents soil physical and chemical properties and Cd, vertical row represents microbial community abundance information, red represents positive correlation, blue represents negative correlation, darker color indicates higher correlation, p value is correlation test result, * in the figure indicates *p*<0.05, ** indicates *p*<0.01, and *** indicates *p*<0.001. Total soil carbon (C); Total soil nitrogen (N); Total soil phosphate (P); Total soil kalium (K); Soil organic carbon (SOC); Available soil phosphate (AP); Available soil potassium (AK); Total soil cadmium (TCd); Available soil cadmium (ACd)

In contrast, in soils with increased cadmium concentrations, the change in the relative abundance of *Acidobacteria* (5.00%) was higher than those of *Saccharibacteria* (3.53%) in the CK group. However, the relative abundance of *Saccharibacteria* (average 4.81, ranging from 3.41% to 6.17%) was higher than that of *Acidobacteria* (average 3.67%, ranging from 2.88% to 4.36%) in the cadmium-addition treatment groups, and the composition of *Chloroflexi* and *Gemmatimonadetes* was reversed between the CK group and cadmium-addition treatment grous. Similarly, in the fungal species, the fungal composition of *Glomeromycota* and *Mortierellomycota* showed the opposite trend. These results were further confirmed by Spearman's rank correlation coefficients analysis, as shown in Fig. 6.

**Fig. 7:**
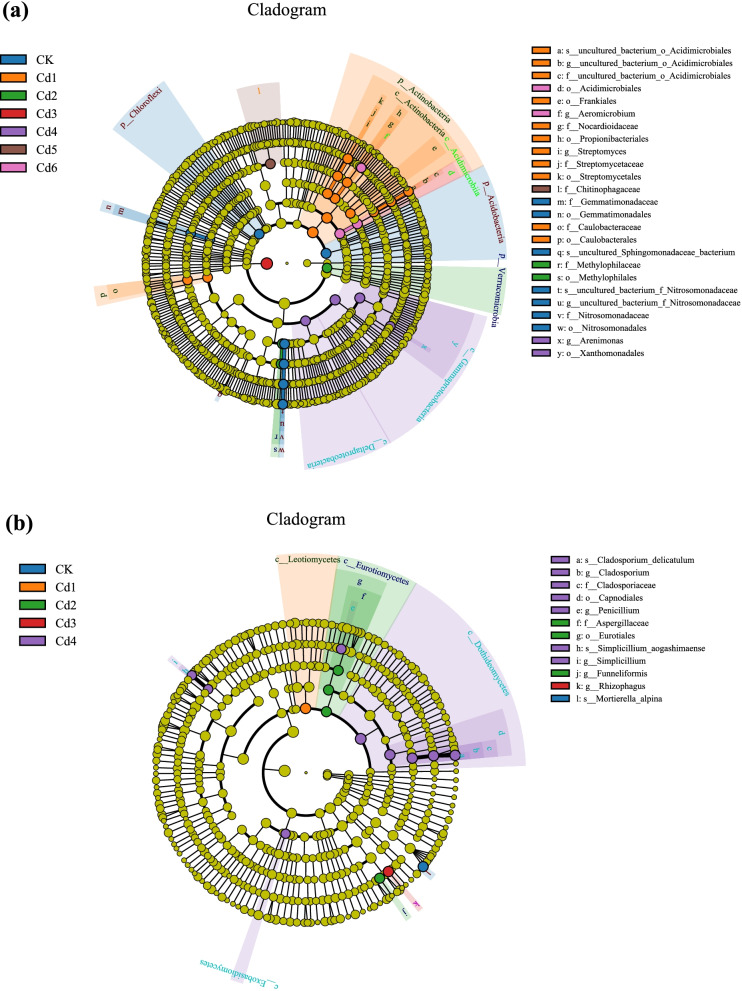
Phylogenetic dendrogram of biomarker bacterial (**a**) and fungal (**b**) among all Cd addition samples

To further study the effects of added Cd in soil microbial abundance and composition, the linear discriminant analysis effect size (LEfSe) method was used to identify significant genes in all samples based on the Wilcoxon rank sum test. A phylogenetic dendrogram of biomarker bacteria (BmB) and fungi (BmF) for different Cd-added treatment samples is shown in Fig. 7. In total, 45 differentially abundant bacterial abundant taxonomic clades and 16 differentially abundant fungal taxonomic clades were identified with an LDA score higher than 4.0.

## Discussion

### Cd addition changes soil microbial biodiversity

A previous study indicated that Cd addition can cause a decrease in pH and affect the available nutrient supply [29]. In this pot experiment, Cd addition directly increased the total and available Cd contents in the soil. The available Cd content in the Cd6 group was more than 91 times than in the CK group (Fig. 1). The addition of Cd significantly reduced the soil pH and inhibited the supply of available NPK in the soil (Fig. S1). These results are probably due to the fact that soil dissolves organic matter, mainly soil humus, protein, fatty acids, amino acids and citric acid, and this effect is gradually fixed by the excessive available Cd [30].

Both bacterial and fungal alpha diversity were, to some extent, correlated with soil physicochemical properties, especially in the treatment groups with Cd added. These finding suggest that high Cd concentrations affected biodiversity. Our experiments support the hypothesis that Cd contamination (treatments from Cd1 to Cd6) affects the species richness and diversity of soil microbes, especially soil bacteria. Previous studies indicated that soil microorganisms were highly sensitive to stress caused by heavy metals because these contaminants significantly affected their growth and metabolism through functional disturbances, protein denaturation, or cell membrane destruction [31–33]. The ACE and Chao1 indices were used for microbial richness, and the Shannon index reflects microbial diversity [34]. Our results showed that the Shannon index of the soil bacteria in the Cd1 treatment group was lower than that in the other treatment groups (*p*<0.01 or *p*<0.001). The ACE and Chao1 indices showed the same trend as the Shannon index (Fig. 2), but the Shannon, ACE, and Chao1 indices of the higher Cd treatment groups (from Cd2 to Cd6) were not obviously different from those of the CK group. Although the Cd concentration significantly affected soil bacterial richness and diversity, there were few effects in fungi, suggesting that the effect of Cd contamination on bacterial diversity was greater than that on fungal diversity. This result is consistent with the findings of other researchers [35, 36]. One possible reason is that soil microorganisms adapt to the stress of low Cd concentrations by regulating their physiological metabolism. However, some microbial communities may be sustained and multiply even in the presence of high Cd concentrations. Contaminants can alter some local microbial populations by exerting selective pressure but leave other populations intact [27].

### Microbial community composition and key species

Given that both microbial abundance and community composition were highly affected by the Cd contamination, the Spearman's rank correlation coefficients that responded positively to Cd contamination could be groups of taxa that can be rapidly multiplicated (Figs. 2, 3 and 5). the response of the soil microbial community to Cd contamination has been reported in many species, but microbial communities and composition are regulated by many factors, such as heavy metal types and concentrations and plant root exudates [37–39]. Soil microbes are an indicator of soil fertility and participate in material transfer and energy exchange, which are usually considered the link between soil and plants [31, 40]. Many studies have confirmed that heavy metal pollution affects microbial abundance, especially microbial assembly and changes in functional microbial community composition [41, 42]. Different concentrations of Cd addition to soils changed the composition and assemblage of functional microbial communities (Figs. 3 and 4). The results indicated that *Acidobacteria* and *Chloroflexi* in bacteria and *Glomeromycota* in fungi were the Cd tolerant microbial communities, and their assemblage could grow normally in the Cd contaminated soil. In addition, the LEfSe results showed that the Cd1 treatment group had the highest number of biomarker bacteria, followed by the CK, Cd4, Cd2, Cd3, and Cd5. Conversely, the Cd4 treatment had the highest amount of biomarker fungi, followed by CK, Cd2, Cd3, Cd1. The results also explained that the Cd-tolerant taxa was promoted and Cd sensitive taxa was disappeared, which may be associated with the differences of microbial function, such as their abilities to absorb nutrients and metabolism differ [43].

A previous study indicated that heavy metal contamination leads to changes in the community structure and function [44–46]. The reorganization of the soil microbial community formed a new microbial assembly and changed its relevant function, which may be an adaption for its ecological services in Cd-contaminated soil. A significant decrease in bacterial diversity was found in the treatment groups with higher concentrations of Cd addition in ginseng-growing soil, and this was associated with significant changes in bacterial and fungal composition (Figs. 2 and 5). The possible reason for this is that the number of Cd-sensitive organisms was reduced, so the number of resistant organisms increased in the ginseng growing soil. This could lead to changes in diversity and composition. In addition, sequences belonging to members of the *Acidobacteria* and *Chloroflexi* phyla are commonly found in many heavy metal- contaminated soils, including mercury-stressed soils [47], farmland soils [48], sediment soils [49], and Cd/As-contaminated soils [50]. Similarly, in the fungal community, the *Glomeromycota* community can survive in Cd-contaminated soil. One possible mechanism is that arbuscular mycorrhizal fungi (AMF) belong to *Glomeromycota*, and AMF have been shown to increase the tolerance of plants to the presence of heavy metals [51, 52]. These shifts in bacterial and fungal community life strategies are significant in predicting the responses of microorganisms and soil biogeochemical cycling to changes in ginseng-growing soils polluted with Cd contamination.

### Environmental drivers of microbial communities

Soil microbial community structure is highly sensitive to soil environmental changes and stresses [53, 54] and is extremely affected by heavy metals [55–57]. Our results in Cd-contaminated ginseng-growing soil (Figs. 3 and 5) and a recent analysis of agricultural paddy soil microbial community assembly [58] support that the influence of deterministic processes might cause dominant microbe composition changes and increase Cd-tolerant microorganism assembly. Moreover, the bacterial diversity was in agreement with the findings of a previous study [59]. A low concentration of heavy metals promoted bacterial reproduction, but high concentrations led to a reduced bacterial population.

As expected, soil bacterial diversity was markly affected by Cd addition treatments, although there was no significant difference in fungal diversity. These findings indicate soil bacteria were more sensitive to Cd contamination than fungi. This result is consistent with the results from a study on saline-alkaline stress in Cd-contaminated soil [60]. Although Cd concentration is a dominant factor for shaping soil bacterial communities, other soil factors, such as pH, OM, and available NPK, are often even more important because they can reduce the toxicity of Cd contamination [61]. CDA indicated that total and available Cd, AK, AP, pH, NH_4_^+^-N, and OM significantly affected soil bacterial and fungal communities, and the environmental factors may be the most important factors explaining both bacterial and fungal community compositions (Fig. 4). A heatmap of Spearman's rank correlation further supported our third hypothesis that some functional microbial groups, such as *Verrucomicrobia*, *Armatimonadetes*, *Proteobacteria*, *Parcubacteria*, *Saccharibacteria* and *Bacteroidetes* in bacteria and *Glomeromycota* in fungi, were significantly positively correlated with total and available Cd, but significantly negatively correlated with AK, AP and pH. The significant positive correlation between Cd and the key microbes further indicated that microorganisms might generate resistance and enable themselves to survive by increasing the transport of metal ions and excreting metal ions. These results were confirmed by previous studies in bamboo plantations [62], activated sludge systems [63] and smelter-contaminated soil [64]. One possible mechanism may be that taxa are generally considered a dominant part of the metabolically active bacterial community in heavy metal-contaminated soil [65].

These dominant phyla could adapt to extremely contaminated soil. For example, *Verrucomicrobia* and *Proteobacteria* are oligotrophic bacteria that contribute to predicting the multifunctional resistance of ecosystems, especially for heavy metal resistance [32]. Actinobacteria can produce a variety of extracellular hydrolytic enzymes that degrade soil organic compounds and enhance the cycling of C, N, and other elements. Similarly, *Glomeromycota* belong to the fungal community, which is the tolerant to the presence of heavy metals [64].

## Conclusion

Cd contamination not only affected soil pH and available NPK, but also shaped microbial diversity and community composition under pot culture conditions in Cd-contaminated ginseng-growing soil. Using Illumina HiSeq rDNA sequence analysis, we found that Cd contamination affected bacterial diversity more than fungal diversity. Additionally, Cd contamination significantly changed the composition of the soil bacterial and fungal communities but had no significant impact on the species of the dominant microbes. Specifically, *Saccharibacteria*, *Gemmatimonadetes* and *Mortierellomycota* may be microbial factors that can monitor microorganisms during the aggravation of Cd contamination. In addition, some key biomarkers were identified at different classification levels in Cd-contaminated soil, which can also be regarded as Cd-contaminated monitoring factors. In the future, the potential application prospects of these tolerant biomarkers (BmB of BmF) should be explored to find the most suitable habitat conditions under different levels of Cd contamination. This could fully expose their unique heavy metal transfer and absorption functions to provide the corresponding reference basis for bioremediation in Cd-contaminated ginseng regions.

## Methods

### Culture and experimental design

The experiment was conducted in an intelligent cultivation center. The room temperature, light intensity and humidity cultivation were controlled at the Institute of Special Animal and Plant Sciences of CAAS, Changchun, China. These cultures were submitted to a photoperiod of 12 h:12 h light/dark with constant aeration, and the light intensity was set at 95 μmol·m^-2^·s^-1^. The temperature of the cultures was kept at 21 °C by controlling room temperature, and the humidity was set at 65% during the experiment.

A pot culture experiment based on a completely randomized design (CRD) was conducted from October 2018 to March 2019 at the Institute of Special Animal and Plant Sciences of CAAS, Changchun, China. The dimensions of the black PVC pot were as follows: bottom diameter of 130 mm and height of 180 mm. The experiment was set up to have seven treatments with three replicates. The pot soil was collected from a three-year abandoned farmland in the town of Changchun City of Jilin Province (E:125°26'24", N:43 °46'24"). The soil texture was sandy loam with 51.26% of sand, 7.67% of clay and 41.07% of silt based on the international soil society classification of soil texture. The basic soil properties were as follows: the pH was 6.64, and organic matter, total phosphate, total potassium, NH4+-N, NO3--N, available phosphate and available potassium were 4.05 g kg-1, 0.98 g kg-1, 14.81 g kg-1, 36.37 mg kg-1, 3.98 mg kg-1, 14.98 mg kg-1 and 270.38 mg kg-1, respectively. In each pot, a piece of nylon filter (130 cm^2^) with a mesh diameter of 55 mesh was placed at the bottom and then it was filled with 3.0 kg dry soil mixed with different cadmium concentrations. The treatments were as follows: (CK) no cadmium addition, (Cd1) 0.25 mg cadmium per kg dry soil, (Cd2) 0.50 mg cadmium per kg dry soil, (Cd3) 1.00 mg cadmium per kg dry soil, (Cd4) 2.00 mg cadmium per kg dry soil, (Cd5) 5.00 mg cadmium per kg dry soil, and (Cd6) 10.00 mg cadmium per kg dry soil. Each treatment was replicated three times, and each pot was considered a replicate. One week after the Cd was added, five three-year-old ginseng seedlings with consistent weights, shapes, and sizes were chosen and cultivated, and the water content was controlled by weight. Irrigation was conducted if the water content was lower than 18% (60% of FC) during the experiment.

### Soil sample preparation

Approximately 110 d of cultivation after the ginseng was planted, fresh rhizosphere soil samples were collected from ginseng roots after the ginseng from one sample withered. A total of 21 samples were collected and immediately passed through a 2 mm sieve. Then, the treated soil was divided into two subgroups: air-dried for chemical property analysis and stored at -80 °C for DNA extraction.

### Analysis of soil properties

Total carbon (TC) and total nitrogen (TN) were determined using an element analyser (Vario EL, Germany). Soil organic matter (SOM) was measured via a potassium dichromate oxidation method [66]. Soil pH was measured at a 1:5 (soil:water) ratio with a glass electrode (FE20, Switzerland). Inorganic N (NH_4_^+^-N and NO_3_^-^-N) was determined on a continuous flow analyser (AA3, Germany) after extraction from soil with 2 M KCl. Available phosphorus (AP) and available potassium (AK) were analysed by the analysis method of soil agricultural chemistry [67]. Total phosphate (TP) was measured with molybdenum blue using spectrophotometry (T6, China). Total potassium (TK) was measured using a flame photometer (6400A, China). Total and available Cd concentrations were measured by full spectrum direct-reading inductively coupled plasma emission spectrometry (Varian 710ES, American) based on a previously described method [68].

### DNA extraction, PCR amplification and sequencing

Soil DNA was extracted based on the manufacturer's instructions of the Power Soil DNA Isolation Kit (MO BIO Laboratories). DNA quantification and PCR amplification were performed as described in a previous study [5]. Each soil sample was extracted three times and then mixed and sequenced. High-throughput sequencing analysis of bacterial/fungal rDNA was performed using the Illumina HiSeq 2500 platform (2×250 paired ends) at Biomarker Technologies Corporation, Beijing, China.

### Bioinformatics analysis

The microbial sequence analyses were completed on the Biomarker biocloud platform (www.biocloud.org). The paired-end reads were merged by FLASH (v1.2.7, http://ccb.jhu.edu/software/FLASH/) [69], and the treated raw tags were filtered and clustered in the next steps. Finally, the tags with an average quality score <20 in a 50 bp sliding window were abandoned using Trimmomatic (http://www.usadellab.org/cms/?page=trimmomatic) [70] and tags shorter than 350 bp were removed. The possible chimaeras were identified by employing UCHIME, which is a tool included in mothur (https://drive5.com/usearch/manual/uchime_algo.html). The denoised sequences were clustered using USEARCH (version 10.0) and tags with similarity ≥97% were regarded as OTUs. Bacterial taxonomy was assigned to all OTUs by searching against the Silva databases (Release128, http://www.arb-silva.de.) using the uclust within QIIME. The fungal taxonomic information was identified using the UNITE database (https://unite.ut.ee/) based on the BLAST tool in QIIME (version 1.9.1) [71].

### Data analysis

The Shannon index, ACE and Chao 1 data and fungal taxa abundance were analysed using GraphPad Prism 5.0 (GraphPad Software Inc., San Diego, CA, USA), and the significant differences in the microbial alpha diversity between different cadmium treatments were calculated using Duncan's multiple test at *p*<0.05, *p*<0.01 or *p*<0.001, respectively. Total and available cadmium were calculated using SAS version 9.1(SAS Institute Inc., Cary, NC, USA). One-way-analysis of variance (ANOVA) and Spearman's correlations were also calculated using SAS version 9.1(SAS Institute Inc., USA). To determine the potential differences in the bacterial and fungal communities across compartments and chromium-added treatments, a heatmap of Spearman's rank correlation was generated using the package “gpplot” (Oksanen et al., 2012) in R (v3.6.2) based on Bray-Curtis dissimilarity. Canonical discriminant analysis (CDA) was performed to calculate the environmental factors that have the most remarkable influence on the microbial and fungal communities by CANOCO 5.0 (Biometrics Wageningen, Netherlands) [72, 73]. LEfSe analysis was applied using the version at https://huttenhower.sph.harvard.edu/galaxy/ [74].

## Supplementary Information

**Additional file 1.**

## Data Availability

The datasets supporting the conclusions of this article are available in the NCBI repository [unique persistent identifier and hyperlink to datasets in https://www.ncbi.nlm.nih.gov/sra/PRJNA714690; accession project number: PRJNA714690; and https://www.ncbi.nlm.nih.gov/sra/PRJNA714699; accession project number: PRJNA714699;].

## Abbreviations

Cd: Cadmium
SOM: Soil organic matter
TN: Total nitrogen
TP: Total phosphate
TK: Total potassium
AP: Available phosphate
AK: Available potassium
CDA: Canonical discriminant analysis
CRD: Completely randomized design
ANOVA: Analysis of variance
UPGMA: Unweighted Pair Group Method with Arithmetic mean
NCBI: National Center for Biotechnology Information
