# Supplementary material for: Effects of cadmium contamination on bacterial and fungal communities in Panax ginseng-growing soil
Source: BMC Microbiol. 2022 Mar 19;22:77. doi: 10.1186/s12866-022-02488-z (PMC8933969; doi:10.1186/s12866-022-02488-z)
Supplement: Supplementary file 1 — Additional file 1. [file 12866_2022_2488_MOESM1_ESM.docx]

**Supplementary Information:**

Fig. S1 Canonical discriminant analysis biplots to investigate the ecological correlation between abundance of key biomarker and characteristics.


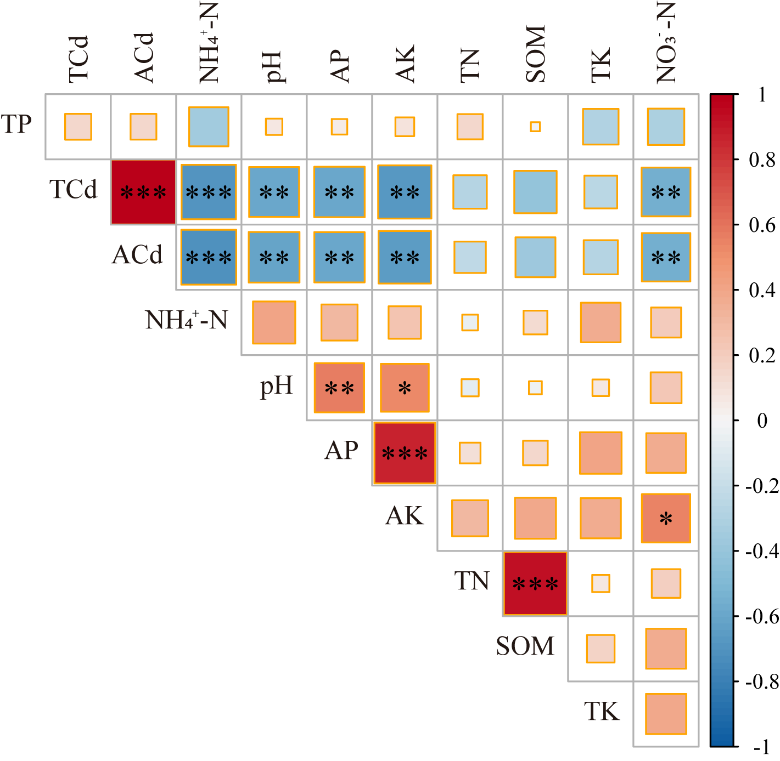


Table S1. The results statistic of soil bacterial sequencing data

| Treatments | Replication | PE reads | Raw Tags | Clean Tags | Effective Tags | Average Length(bp) | GC(%) | Q20(%) | Q30(%) | Effective(%) | OTUs |
| --- | --- | --- | --- | --- | --- | --- | --- | --- | --- | --- | --- |
| CK | CK1 | 78115 | 68,932 | 55,667 | 55,142 | 418 | 54.33 | 93.2 | 87.23 | 70.59 | 892 |
|  | CK2 | 66423 | 58,839 | 47,265 | 47,046 | 419 | 55.51 | 93.26 | 87.25 | 70.83 | 800 |
|  | CK3 | 63310 | 56,822 | 46,203 | 45,807 | 418 | 54.24 | 93.28 | 87.35 | 72.35 | 871 |
| Cd1 | Cd1-1 | 56609 | 51,448 | 47,448 | 46,969 | 417 | 55.01 | 95.11 | 90.25 | 82.97 | 804 |
|  | Cd1-2 | 46426 | 41,595 | 38,010 | 37,677 | 417 | 54.84 | 94.85 | 89.79 | 81.15 | 763 |
|  | Cd1-3 | 48346 | 43,706 | 40,237 | 39,066 | 416 | 54.73 | 95.05 | 90.12 | 80.81 | 774 |
| Cd2 | Cd2-1 | 55366 | 49,839 | 45,594 | 45,243 | 419 | 54.52 | 94.98 | 90.09 | 81.72 | 887 |
|  | Cd2-2 | 53046 | 47,886 | 43,657 | 43,307 | 419 | 54.73 | 95.01 | 90.07 | 81.64 | 894 |
|  | Cd2-3 | 53985 | 48,813 | 44,707 | 44,435 | 419 | 54.62 | 95.02 | 90.12 | 82.31 | 830 |
| Cd3 | Cd3-1 | 80060 | 72,633 | 66,718 | 66,160 | 417 | 54.92 | 95.04 | 90.2 | 82.64 | 901 |
|  | Cd3-2 | 75641 | 68,615 | 63,187 | 61,701 | 417 | 54.78 | 95.03 | 90.18 | 81.57 | 896 |
|  | Cd3-3 | 80000 | 72,866 | 66,862 | 66,113 | 418 | 54.89 | 95.04 | 90.19 | 82.64 | 918 |
| Cd4 | Cd4-1 | 62324 | 56,098 | 51,163 | 50,721 | 418 | 54.43 | 94.9 | 89.97 | 81.38 | 905 |
|  | Cd4-2 | 57391 | 52,050 | 47,676 | 47,136 | 418 | 54.58 | 94.99 | 90.11 | 82.13 | 919 |
|  | Cd4-3 | 77097 | 69,548 | 63,783 | 63,155 | 418 | 54.8 | 95.03 | 90.19 | 81.92 | 922 |
| Cd5 | Cd5-1 | 72388 | 65,591 | 60,221 | 59,609 | 418 | 54.51 | 95.04 | 90.22 | 82.35 | 904 |
|  | Cd5-2 | 79877 | 72,590 | 66,436 | 65,884 | 418 | 54.51 | 95.01 | 90.15 | 82.48 | 910 |
|  | Cd5-3 | 79659 | 72,886 | 67,528 | 66,339 | 417 | 54.68 | 95.29 | 90.63 | 83.28 | 918 |
| Cd6 | Cd6-1 | 68446 | 62,692 | 58,271 | 57,497 | 417 | 54.73 | 95.3 | 90.66 | 84 | 883 |
|  | Cd6-2 | 79876 | 73,425 | 68,263 | 66,863 | 417 | 54.85 | 95.29 | 90.61 | 83.71 | 886 |
|  | Cd6-3 | 68518 | 60,406 | 55,896 | 55,047 | 417 | 54.86 | 95.13 | 90.36 | 82.76 | 896 |

Table S2. The results statistic of soil fungal sequencing data

| Treatments | Replication | PE reads | Raw Tags | Clean Tags | Effective Tags | Average Length(bp) | GC(%) | Q20(%) | Q30(%) | Effective(%) | OTUs |
| --- | --- | --- | --- | --- | --- | --- | --- | --- | --- | --- | --- |
| CK | CK1 | 80,060 | 67,912 | 65,983 | 65,968 | 248 | 41.02 | 97.66 | 94.82 | 82.4 | 180 |
|  | CK2 | 80,025 | 69,150 | 67,129 | 66,866 | 245 | 40.73 | 97.95 | 95.29 | 83.56 | 211 |
|  | CK3 | 79,985 | 63,568 | 61,650 | 61,344 | 255 | 42.38 | 97.49 | 94.5 | 76.69 | 149 |
| Cd1 | Cd1-1 | 80,020 | 67,285 | 65,337 | 65,067 | 234 | 45.62 | 98.45 | 96.2 | 81.31 | 136 |
|  | Cd1-2 | 79,753 | 65,714 | 63,786 | 63,647 | 231 | 44.84 | 98.43 | 96.17 | 79.81 | 178 |
|  | Cd1-3 | 77,960 | 64,682 | 62,844 | 62,681 | 233 | 43.7 | 98.35 | 96.04 | 80.4 | 192 |
| Cd2 | Cd2-1 | 79,951 | 67,442 | 65,285 | 64,857 | 238 | 45.77 | 98.29 | 95.91 | 81.12 | 307 |
|  | Cd2-2 | 79,616 | 67,151 | 65,154 | 64,416 | 240 | 42.97 | 98.14 | 95.67 | 80.91 | 187 |
|  | Cd2-3 | 80,101 | 67,886 | 65,759 | 65,649 | 241 | 43.05 | 98.13 | 95.67 | 81.96 | 164 |
| Cd3 | Cd3-1 | 79,788 | 66,497 | 64,609 | 64,465 | 242 | 43.91 | 98.09 | 95.56 | 80.8 | 183 |
|  | Cd3-2 | 80,366 | 67,344 | 65,356 | 65,274 | 241 | 42.24 | 98.02 | 95.47 | 81.22 | 169 |
|  | Cd3-3 | 79,820 | 65,353 | 63,466 | 63,326 | 241 | 41.74 | 98.01 | 95.45 | 79.34 | 161 |
| Cd4 | Cd4-1 | 80,155 | 67,138 | 65,340 | 65,032 | 232 | 43.13 | 98.31 | 95.96 | 81.13 | 180 |
|  | Cd4-2 | 80,227 | 66,123 | 64,310 | 63,868 | 232 | 45.46 | 98.71 | 96.61 | 79.61 | 162 |
|  | Cd4-3 | 80,177 | 66,526 | 64,647 | 64,039 | 232 | 43.37 | 98.28 | 95.92 | 79.87 | 162 |
| Cd5 | Cd5-1 | 80,225 | 66,310 | 64,337 | 63,560 | 244 | 40.82 | 97.69 | 94.95 | 79.23 | 160 |
|  | Cd5-2 | 79,829 | 66,168 | 64,211 | 63,452 | 235 | 43.88 | 98.38 | 96.07 | 79.48 | 147 |
|  | Cd5-3 | 79,914 | 69,500 | 67,453 | 66,478 | 238 | 41.4 | 98.17 | 95.81 | 83.19 | 180 |
| Cd6 | Cd6-1 | 80,418 | 68,992 | 67,108 | 66,290 | 224 | 41.17 | 98.52 | 96.43 | 82.43 | 207 |
|  | Cd6-2 | 80,219 | 68,235 | 66,097 | 65,589 | 256 | 39.13 | 97.33 | 94.41 | 81.76 | 163 |
|  | Cd6-3 | 80,191 | 68,801 | 66,819 | 65,853 | 234 | 41.16 | 98.3 | 96.05 | 82.12 | 181 |
